# Flourishing, Mental Health Professionals and the Role of Normative Dialogue

**DOI:** 10.1007/s10728-023-00478-4

**Published:** 2024-01-12

**Authors:** Hazem Zohny, Julian Savulescu, Gin S. Malhi, Ilina Singh

**Affiliations:** 1 Oxford Uehiro Centre for Practical Ethics, University of Oxford, Oxford, UK; 2 Department of Psychiatry, University of Oxford, Oxford, UK; 3 Wellcome Centre for Ethics and Humanities, University of Oxford, Oxford, UK; 4 Centre for Biomedical Ethics, Yong Loo Lin School of Medicine, National University of Singapore, Queenstown, Singapore; 5 Murdoch Children’s Research Institute, Melbourne, Australia; 6 University of Melbourne, Melbourne, Australia; 7 Academic Department of Psychiatry, Kolling Institute, Northern Clinical School, Faculty of Medicine and Health, The University of Sydney, Sydney, Australia; 8 CADE Clinic and Mood-T, Royal North Shore Hospital, Northern Sydney Local Health District, St Leonards, NSW, Australia; 9 Visiting Professor, Department of Psychiatry, University of Oxford, Oxford, Australia; 10 Oxford Uehiro Centre for Practical Ethics, Faculty of Philosophy, University of Oxford, Oxford, Australia

**Keywords:** Mental healthcare, Patient well-being, Flourishing, Liberal approaches, Values-based practice, Shared-decision making

## Abstract

This paper explores the dilemma faced by mental healthcare professionals in balancing treatment of mental disorders with promoting patient well-being and flourishing. With growing calls for a more explicit focus on patient flourishing in mental healthcare, we address two inter-related challenges: the lack of consensus on defining positive mental health and flourishing, and how professionals should respond to patients with controversial views on what is good for them. We discuss the relationship dynamics between healthcare providers and patients, proposing that ‘liberal’ approaches can provide a pragmatic framework to address disagreements about well-being in the context of flourishing-oriented mental healthcare. We acknowledge the criticisms of these approaches, including the potential for unintended paternalism and distrust. To mitigate these risks, we conclude by suggesting a mechanism to minimize the likelihood of unintended paternalism and foster patient trust.

## Introduction

What do mental healthcare professionals ultimately aim for in the treatment of their patients – is it only to treat mental disorders or to promote their well-being and help them flourish? The two are clearly linked; mental disorders are conditions that reliably diminish well-being. Treating them effectively reliably improves it. But treatment and well-being can come apart. Some treatments come with significant trade-offs. For instance, antidepressant medication can reduce some individuals’ interest in sex [1], while commonly prescribed drugs such as propranolol can impact pro-social behaviour [2]. These treatments may directly or indirectly reduce an individual’s overall well-being despite treating a mental disorder or reducing its symptoms. In some cases, clinicians work with patients to figure out what trade-offs are acceptable given their particular circumstances.

This may seem obvious and true of medical decision-making more generally. However, there are mounting calls in relation to mental healthcare in particular to make this end goal (i.e. promoting patients’ well-being) more explicit and central to clinical decision-making. Positive psychiatry proponents, for instance, argue that clinicians should evaluate not just symptoms and diagnoses, but also patients’ overall well-being, including whatever psychosocial characteristics are associated with flourishing (e.g. resilience, optimism) [3–6]. This expanded remit opens the door to not just treating the psychological impediments to well-being (e.g. mental disorders), but to potentially enhancing the traits associated with flourishing [3].

Put concretely, traditional mental health provision often zeroes in on symptom relief: a depressed teen might receive SSRIs and cognitive therapy, an elderly patient grappling with chronic health conditions might be given anxiety medication, and a schizophrenia patient primarily relies on antipsychotics. In contrast, mental healthcare that focuses on flourishing may go beyond this. It emphasizes enhancing patients’ overall well-being by, for instance, integrating strengths and resilience exercises for the teen, lifestyle and social measures for the elderly, and broad recovery goals alongside medical treatment for the schizophrenia patient. While many elements of this flourishing-orientation overlap with what competent mental health providers already practice, the key distinction lies in its systematic and explicit emphasis on positive aspects of human experience. It integrates principles of positive psychology in a structured way, making the cultivation of well-being and personal strengths its core focus, rather than just a supplemental component of traditional therapy.

There are many questions we could raise in response to these calls, from what this might mean for how we fund access to mental healthcare, to whether they transgress the proper scope of medicine. However, this paper will not attempt to address all challenges (though see [7]) but only two specific inter-related ones.

The first challenge is this: while there may be broad agreement on what constitutes some mental disorders and about the importance of treating them, there is comparatively less agreement about what might constitute *positive* mental health, let alone well-being or flourishing more generally.

The second is this: even if there were broad agreement on the nature or constituents of well-being, how should mental health professionals respond to competent patients who have divergent views about what is good for them? Another way of putting this second challenge is: how should we respond to patients with unusual, controversial, or perplexing conceptions of flourishing, and who wish to have their mental healthcare provider help them realise those conceptions?^1^

We unpack these two challenges in greater detail in Sect. 2. Section 3 introduces a debate on relationship dynamics between healthcare providers and patients which we argue can be instructive to responding to these challenges. We argue that, despite the significant criticisms of some of what we call ‘liberal’ approaches (Sect. 4), they can offer a pragmatic framework for addressing disagreements about well-being in the context of flourishing-oriented mental healthcare provision. Finally, in Sect. 5, we consider the prospect of unintended paternalism and distrust seeping into these liberal approaches, and propose a mechanism to minimize the likelihood of that happening.

Before proceeding, a note on terminology and scope. As is already evident, we have been using the terms ‘well-being’, ‘flourishing’, and a ‘good life’ interchangeably. This is intentional. Common usage suggests these terms are typically deployed to distinguish the subject matter from the notion of mere ‘happiness’. However, even a simple hedonistic (i.e. pleasure-based) account of well-being may cohere with, say, an Aristotelian understanding of the good life, at least to the extent that the associated virtues are a reliable way to maximize happiness and pleasure. Which is to say, regardless of the term used, there will be significant overlap about what is being described.

In terms of scope, we constrain our focus on the individual. While mental health and well-being are entangled with one’s cultural and social context, our interest here is in clinical practice, and the prospect of its re-orientation to a focus on flourishing. We take it that clinical practice – as it currently stands – typically has the individual patient at its heart.

We also take it that clinical practice^2^ may specifically be psychiatric, or it may be broader psychotherapeutic intervention. Since our interest is in the prospect of promoting flourishing through changes to patients’ psychology broadly construed, we will attend to mental healthcare provision more broadly, which we take to include psychiatry. We henceforth use the term ‘therapy’ for that purpose, and ‘flourishing-oriented therapy’ for the view that the promotion of patient flourishing should be the explicit end goal of therapy.

## Two Challenges

As noted in the introduction, we limit the scope of our concern here to two interrelated challenges facing flourishing-oriented therapy: (1) The relative lack of agreement about what makes for a good life, and (2) even if we can reach broad agreement about the elements associated with flourishing, what should therapists do about competent patients who do not subscribe to that agreement? Our goal in this section is to delve into and unpack these two challenges to set up the need for a proposed solution in the next section. Both challenges link to an objection that the concept of flourishing is highly indeterminate, and therefore a poor guide to clinical decision-making.

This objection holds that, while what counts as a ‘mental disorder’ may be contested, surely what counts as a good life is all the more disputable. That is, even though the debate on the nature of mental disorders is vast and far from settled [e.g. 8–10], by and large most will agree that clearly some mental conditions significantly hamper individuals’ well-being in uncontroversial ways. We need not settle the precise ontology or classification of those conditions to agree about that.

In contrast, the scope for disagreement about what makes for a good life, as opposed to merely not a bad one, is vaster. While most might agree chronic depression significantly interferes with leading a good life, they may well disagree about what positive, optimal mental states or conditions look like. Here, the multitude of religious and political views, and the individual’s own desires and goals and social context, create a greater range for disagreement.

Proponents of flourishing-oriented therapy (hereafter ‘proponents’) may respond along two lines. First, they may argue that the agreement about the badness of certain mental conditions is an important starting point. There is in fact broad agreement about what significantly interferes with flourishing (however loosely defined), including the ability to take up opportunities to that end: feeling consistently miserable or fatigued; being gripped by unpredictable mood swings or controlled by obsessive-compulsive behaviours; being highly unmotivated; lacking capacity to reflect on one’s self and actions, and so on.

Proponents could argue that is enough agreement to establish a basis for much of flourishing-oriented therapy. This would characterise the treatment of these conditions as a means to mitigating suffering and, thereby, promoting flourishing, or the capacity to take on opportunities associated with it.

Going beyond that, however, they may also argue that there is significant overlap among the different ways we think about what makes for a good life, rather than merely not a bad one. This seems true for any plausible constituent or contributor to a good life, as well as any philosophical account of it.

In terms of the contributors, attempts to measure flourishing have included, for instance, these six domains: happiness and life satisfaction, mental and physical health, meaning and purpose, character and virtue, close social relationships, and financial and material security [5]. Each of these domains appear nearly universally desired and strongly identified with leading a good life [11]. If there are psychological traits that are highly predictive of being able to succeed in these domains, those too would create a foundation for a flourishing-oriented therapy that goes beyond disorder treatment and prevention.

Moreover, philosophically, there is significant convergence among the main accounts of well-being: hedonism, desire-satisfaction, and objective list theories [12]. Broadly, and somewhat crudely, these refer to understanding well-being as maximizing pleasure and minimizing pain, where pleasure is the only intrinsic good (hedonism); as satisfying desires whether or not doing so gives one pleasure (desire-satisfaction); or as a particular combination of intrinsic goods such as knowledge, friendship and autonomy (objective lists). To see how they converge, consider the examples of friendship, autonomy and knowledge: hedonism and desire-satisfaction theories typically hold these as instrumentally good because, for most people, they give pleasure or satisfy desires. Objective list theories in turn tend to recognize pleasure’s value and typically hold that (at least informed) desires reflect what is independently good.

This is to say that, while these competing accounts might cash out these goods differently (as either intrinsically or instrumentally good, or as well-being’s actual constituents or mere associations), their significant overlap means we need not settle whether there is one true account in order to move forward with well-being or flourishing as therapy’s end goal [13, 14].

Hence, in response to the first challenge that we lack agreement about what makes for a good life to create some foundation for flourishing-oriented therapy, these responses at least minimize the strength of that claim. The problem, however, is that these responses remain unhelpfully vague in relation to individual patients.

Consider the six domains just mentioned [5], or goods such as accomplishment, friendship, pleasure, health, patience. Two individuals may completely agree that these are desirable or even *should* rationally be desirable, yet also wholly disagree about how they would rank them in their lives when they conflict or entail significant trade-offs with each other.

For instance, in some contexts, accomplishment will compete with health. If an individual gives enough weight to health it may come at the cost of accomplishment (e.g. by trying to minimize stresses associated with striving hard). No measure of flourishing or philosophical account tells us how to balance such competing elements in a particular individual’s life. Given how sensitive they are to personal dispositions and broader life circumstances, there may be any number of trade-offs that would be rational for an individual to accept. These theories are not intended to help resolve such personalised trade-offs.

So, the challenge remains: while we may expect agreements to form at the extremes of suffering (e.g. severely disruptive mental conditions), we can expect divergence in cases associated with optimising mental states so as to maximize an individual’s chances of flourishing.

This brings us to the second challenge posed by this objection: what should therapists do when they meet with competent, thoughtful patients who have unusual or controversial conceptions of flourishing? To what extent should therapists prescribe a particular conception of flourishing to their patients, and to what extent should they merely facilitate patients’ own conceptions?

To argue that therapists should adopt or champion a particular conception – i.e. a particular way of weighing up flourishing’s (sometimes) competing constituents or contributors – for their patients, seems to open the door to a degree of paternalism that few are likely to condone, let alone embrace. Therapy already has a history of accusation as an instrument of social control masquerading as medicine [15]. Moreover, any such authoritarian turn that fails to respect patient autonomy risks undermining therapists’ capacity to act in their patients’ best interests due to the likely decline in patient trust – indeed lack of patient involvement in decision-making correlates with lower treatment adherence and lower health outcomes [16–18].

This is a powerful challenge facing flourishing-oriented therapy that has not been addressed. Proponents appear to face a dilemma. At least outside clear-cut cases of severe conditions that uncontroversially interfere with leading a good life, it looks like proponents have to choose between either imposing a particular conception of flourishing onto their patients, or they have to merely act to facilitate their patient’s own conceptions of well-being. Both are unsatisfactory, with one being paternalistic, the other seemingly ‘consumerist’, where the job of therapists is reduced to satisfying their patients’ preferences even if they have good reasons to believe this would lead to outcomes that go against their patient’s overall interests.

In the next section, we home in on this dilemma, and suggest that previous work on the doctor-patient relationship can help proponents of flourishing-oriented therapy to construct an alternative pathway.

## Therapist-patient Relationship Dynamics

One way of reframing this dilemma between authoritarianism and consumerism is by considering more broadly the various relationship dynamics between healthcare providers and their patients. As we will argue, there are different ways of thinking about a patient-centred approach, some more ostensibly consumerist than others. This section aims to propose two “liberal” approaches as a potential solution to the dilemma between authoritarianism and consumerism. It first introduces a framework of doctor-patient relationships and then uses the examples of values-based practice and the liberal rationalist model to describe how therapists may avoid simply imposing or deferring to conceptions of flourishing by eliciting and engaging with patient values through reasoning.

One influential framework provided by Emanuel and Emanuel [19] breaks down doctor-patient relationship options into four approaches: paternalistic, informative, interpretative, and deliberative. Each of these cash out the role of the healthcare provider and patient autonomy differently.

Briefly, a paternalistic approach assigns providers with promoting patients’ interests regardless of their patient’s current preferences, and where patients’ autonomy boils down to assenting to the values the physician is promoting. This approach aligns with what we have been describing as authoritarian. An informative approach sees the provider as a conveyer of relevant factual information, with the patient then using (or failing to use) that information to select an intervention based on their preferences. This aligns with what we have been describing as consumerist. The interpretive approach involves the provider playing a factually informative role but also then helping the patient uncover and interpret their values in light of that information, allowing the patient to make an informed decision in line with that new self-understanding. This approach is still consumerist in the sense that it is ultimately about patient choice, but it crucially takes extra steps to try to minimise the chances that the patient will choose interventions that go against their actual interests. Finally, the deliberative approach builds on the informative and interpretive approach, but also sees the provider as playing the role of a friend or teacher engaging the patient in normative dialogue and actively trying to persuade (though by no means coercing) the patient of the best course of action in light of the interpretive process. Such dialogue is “normative” in that it aims to determine what ought to be, rather than merely what is. This dynamic too boils down to patient choice, but is all the more clearly distinct from reducing healthcare providers to passive purveyors of a service to consumers.

On this framing of our dilemma, there are in fact more choices available to the flourishing-oriented therapist: it need not simply be a choice between authoritarianism or consumerism (though as we will see in the next section critics argue that interpretive and deliberative approaches are nevertheless insidious.). Two examples of approaches that loosely align with an interpretive and a deliberative approach are, respectively, values-based practice (VBP) [20] and the liberal rationalist model (LRM) [21, 22].^3^

VBP is an approach to supporting clinical decision-making rooted in eliciting and understanding patients’ individual values and integrating those into the decision-making process [24]. In that sense it broadly reflects an interpretive approach. LRM, though it has a more general focus on doctor-patient relationships, also involves doctors elucidating patient values. However, LRM argues for a relatively more active role for the doctor to engage the patient in normative dialogue, and to present them with arguments for one course of action over another. In that regard, it is more closely aligned with the deliberative approach.

These two ‘liberal’ models – liberal because they ultimately rest on patient choice – have two premises in particular that should interest flourishing-oriented therapy proponents. The first is that scientific advances are a central motivation for the importance of values (and therefore conceptions of flourishing) in healthcare. Without medical options, there is no role for values and conceptions of well-being to play: if one presents with a condition that is debilitating, there is little discussion to be had about the trade-offs if there is only one way to tackle the condition. Scientific advances generate more ways to medically intervene in the body or mind. As those options increase, the potential trade-offs (e.g. surgery; or drugs with fatiguing side-effects; or talking therapy) come to the fore, and values are more likely to conflict (e.g. reducing symptoms quickly vs. not getting fatigued).

This relates to a second premise of interest, which is that we tend to only notice the role of values in medical decision-making when values conflict. For instance, we typically notice values more when what the patient wants conflicts with what the best evidence suggests will actually improve their mental state, or when the available treatment option entails a conflict between the patient’s own values.

These liberal models are intended to support decision-making in healthcare as we know it – that is, in the diagnosis and treatment of disorders. They are not designed to address a broader flourishing-oriented therapy which may include enhancing mental states. Nevertheless, they offer a way to rethink the dilemma we argued faces proponents in the previous section.

If flourishing-oriented therapy deals not only with mental conditions associated with suffering, but also with optimising the mental states associated with flourishing (be they resilience, optimism, and so on) then we can expect an even larger role for values to play (and conflict). We argued that the options for proponents when met with patients who have unusual or controversial conceptions of flourishing appear to either be an authoritarian prescription of the good life, or to treat them as consumers and abide by their flourishing conceptions (at least so long as those conceptions do not entail treatments that are illegal or against principles of distributive justice medicine^4^).

These liberal models argue for a democratic alternative to authoritarianism and consumerism. Democracies theoretically work *for* the people (in this case patients), but decision-making is still constrained by various constitutional processes. For VBP at least, that process – called ‘good process’ – relates to a framework of skills and commitments in mental healthcare provision [25]. These include being aware of the values and facts relevant to a patient’s specific context; communicating using clear reasoning to explore the values present when making a decision; not applying a ‘pre-prescribed rule’ but working towards finding equilibrium between different perspectives; and approaching decision-making as a partnership.

On the face of it, such an approach appears to differ from a purely informative or, as we have been calling it, ‘consumerist’ approach where therapists only act to help patients realise their own conception of flourishing. While a liberal approach is ‘user-centred’ and ultimately rooted in patient choice, that choice is supposed to be produced via a process of reasoning and value exploration in partnership with the therapist. This means that, rather than shrugging when met with patients who have unusual or controversial flourishing conception, therapists have a role to play in bringing out and examining the underpinnings of that conception, subjecting it to the available evidence where appropriate, and striving to reach a balance between the patient’s (likely inevitable) competing values informing that conception. This is all the more so the case with LRM, where doctors and other health professionals can challenge patient values and engage in normative dialogue, in the same way as a friend might challenge one to consider what *really* matters.

To be clear, no advocate for VBP or LRM would deny that paternalism or a purely informative approach can at times be permissible: no one is arguing that an incoming patient in the midst of a severe psychotic episode needs to have their values elucidated in that moment before some (at least short-term) intervention. Which is to say, nothing about these approaches rules out paternalism in such cases. Similarly, only an ideologue would disregard the fact that resources are limited, and that therapists need the time and skills to deploy the process of uncovering patients’ values or deliberating over them in light of the best evidence. If those resources are lacking, such that providers do not have the time and/or skills to do that, then temporarily adopting an informative approach for otherwise competent, thoughtful patients may be the best of an already bad situation. Accepting this compromise is fully compatible with also holding that these resource limitations may be unjustified.

So far, we have argued these liberal models ostensibly offer proponents of flourishing-oriented therapy a way to respond to the challenge that therapists must either impose a particular conception of flourishing on patients, or treat them merely as consumers. We say ostensibly because critics of these liberal models have argued that, despite the façade of a partnership, they in fact operate on what some call a “neo-liberal” agenda that assumes choice is always good, that saddles patients with responsibility for their choices [26], and that expects clinicians to take an agnostic approach to values [27, 28]. The result, critics argue, is that we conflate good outcomes with this ‘good process’, which at least VBP appears to adopt as an end in itself.

In the next section, we elaborate on and respond to these concerns, and consider their implications for flourishing-oriented therapy.

## Critics of Liberal Models

We began by arguing that flourishing-oriented therapy faces two interrelated challenges: a lack of agreement about flourishing, and a lack of framework for how to respond to patients with unusual or controversial flourishing conceptions. We suggested that, even if we can find broad agreement, it is not clear what this would mean for therapists: do they impose that agreement on patients, or do they retreat to passively provide a service that helps them realise their own conception of a good life? Liberal models, which are designed to varying degrees to negotiate competing values through a ratification process, look like they might help address this dilemma, but they have their critics.

A central criticism is that, while approaches like VBP and LRM present themselves as a democratic approach to decision-making, in fact they still ultimately treat patients as consumers. They treat patients as consumers in that, despite the emphasis on ‘good process’ – to use VBP’s terminology – they are at bottom ‘user-centred’, and unless there are legal or distributive justice constraints, or clear elements of incompetence or danger associated with a patient’s choices, then patient autonomy trumps all. Brecher describes this as “saddling” patients with choice in the sense that, aside from the final choice about treatment options, they are also inundated with choice through the process of uncovering their values and resolving conflicts between them [26].

Equally central to this criticism is that this process of uncovering patient values seems to suggest that clinicians are expected to remain agnostic about the rightness or wrongness, goodness or badness, of those values [27, 28]. This means, at least for VBP or any mostly interpretive approach, clinicians lack the authority to make any substantive judgements about patients’ values. The result is that we end up replacing any commitment to right or good values and outcomes, with a commitment to a right or good process of ratifying them. We then mistake this process as an end in itself, when clearly the end should be what will actually increase the chances of a good outcome for the patient.

In that way, the criticism goes, values are not evaluated in light of any principled stance. The answer to what makes a value judgement true or false becomes a non-sequitur. Instead, a ‘just’ outcome is reduced to having the various values subjected to procedural ratification; so long as the competing views are elicited and an attempt at balance is made, then the outcome is just [27].

These criticisms translate straightforwardly to a flourishing-oriented therapy that takes a similarly liberal approach. If the response to the fact that some patients will have different (and perhaps controversial) conceptions of flourishing is that therapists need to elicit their underpinnings and subject them to reasoning and strive for equilibrium between the competing values, then this seems to equally rely on a perverse notion of what counts as a just or good outcome. So long as patients’ conceptions survive this process, then the outcome is just, and ultimately patient choice reigns. This too, then, would seem to subscribe to, as Brecher [26] puts it, the ‘neo-liberal mantra of choice’ and appears to involve therapists adopting an agnostic view on flourishing that (wrongly) takes the process of ratifying patients’ own conception as an end in itself.

The irony is that some critics, in particular of VBP, have argued that an explicit focus on flourishing would help resolve these problems. According to Hutchinson [28], once we acknowledge the liberal conception of value operating behind the scenes, we can make real progress: “We might then talk of how medical practice should be embedded in a conception of the Good Life or human flourishing (Eudaimonia, as the Greeks called it), for example.” That is, Hutchinson is arguing for what appears indistinguishable from flourishing-oriented therapy as a solution to the problems of a liberal approach, and yet here we are arguing for a liberal approach as a solution for the dilemma facing flourishing-oriented therapy.

One reason for this might be because critics such as Hutchinson appear to have bitten the bullet on the dilemma we presented, and are arguing that therapists should prescribe a particular conception of flourishing on their patients, so long as we are honest about that. For instance, Hutchinson goes on to say: “Sure, we will have arguments over the specific nature of that Good Life, but those are honest arguments where our philosophical commitments are explicit, and where our conception of value does not get smuggled in inside the ‘Trojan horse’ of putatively neutral procedures.” [28].

Proponents of flourishing-oriented therapy are likely to whole-heartedly agree with this – nonetheless, it tells us nothing about what to do when a decision needs to be made [29]. What are critics suggesting therapists should do when these honest arguments nevertheless lead to disagreements between the conclusions reached by therapists and their patients (or patients and their families)? It seems that either it leads to authoritarianism, or the therapist will respect that, even after honest arguments (i.e. a ‘good process’ of some kind), the patient has reached a different conclusion that is worthy of being respected.^5^

However, there is an alternative to interpreting liberal models’ commitment to good process as one of being agnostic about value or conceptions of flourishing. In fact, one may support an approach like VBP without placing any intrinsic value on patient autonomy at all. This is akin to supporting democratic processes without confusing them for being inherently valuable. Indeed, one may think democracy is the worst form of government except – as the saying goes – for all the others that have been tried^6^. In other words, the focus on procedural ratification – doubtless a process that should be amenable to tweaks and updates – may indeed be deeply unsatisfactory in the face of objectively bad outcomes (e.g. when patients continue to subscribe to poor conceptions of flourishing even after that process), but it could still be better than the authoritarian alternative.

There may well be principles by which to appraise value judgements as true or false, but there may also be a principle of pragmaticism that one is committed to that justifies a focus on procedural ratification as the least bad of the alternatives. That is, flourishing-oriented therapy may ultimately lean on patient choice, not because there is anything sacrosanct about autonomy, but because that is the most pragmatic course of action given the authoritarian alternative. This avoids mistaking any ‘good process’ as end in itself. Until critics offer an alternative, or argue convincingly for authoritarianism for otherwise competent patients, this stands as the least bad option, and it is not one that requires being agnostic about values, or the corresponding conceptions of flourishing they relate to.

## Choosing the Right Dynamic

Our response to critics in the preceding section does not tell us whether flourishing-oriented therapy ought to adopt an interpretive approach similar to VBP, or a deliberative one similar to LRM. Our argument so far is that proponents need not confuse a ratification process as an end itself, but simply a pragmatic solution that avoids an authoritarian prescription of a particular conception of flourishing. However, the choice of liberal model can have significant differences and comes with another risk we have so far not discussed, which is that unintended paternalism can seep into these approaches.

If a therapist using VBP or LRM lacks the necessary skills and/or time to help patients articulate their values, the therapist may inadvertently impose their own values while appearing to help the patient express their own [19, 26]. In situations where patients are burdened by their medical condition and feel unsure about their values, they will be susceptible to simply acquiescing to the therapist’s values. In these cases, the dynamic between patient and doctor may unintentionally shift to a more paternalistic approach in practice.

This applies in particular to LRM: patients may be poorly placed to respond to a therapist presenting an argument for taking a particular course of action, even if choice ultimately rests with the patient. This may be because they are overwhelmed by their medical condition, but also because patients typically hold therapists as authoritative even if the goal is shared decision-making. Moreover, the therapist’s presentation of an argument for a particular course of action may not be well received by some patients. In fact, some patients may avoid seeing a therapist altogether if they think they will be confronted with a therapist who advocates for a position that may conflict with their own. For instance, a patient who is strongly opposed to taking antidepressants may avoid seeing a therapist completely if they believe they will be presented with arguments to take the medication, even if they know the choice remains their own at the end.

This is not so much an objection to using liberal models, but a risk that comes with adopting them. As a concluding section, we will not address this risk in great detail, though wish to raise a potential solution that future research may consider more closely. That solution borrows from a similar debate on informed consent, and specifically, on the question of how much information patients should be given in order to provide informed consent. Ludewigs and colleagues argue that patient preferences for the amount of information they wish to receive regarding their diagnosis and treatment options can vary widely [31]. Some patients wish to be informed about every detail of their treatment, while others may prefer to know as little as possible and defer medical decision-making to their physicians. Their proposed solution is to simply ask patients how much information they want to receive about their treatment. This can be done through a brief, preliminary conversation designed to establish the patient’s desired level of information and to clarify what can and cannot be left out.

As with our previous discussion on ‘good process’, the rationale here need not assume that patient choice trumps all other considerations, or even that it is intrinsically valuable so as to outweigh other considerations such as good outcomes. Instead, we could understand the rationale merely as a pragmatic solution to answering the question of how much information is sufficient, given that patients can have highly different expectations and preferences.

For our purposes, we can tweak this proposal and apply it to the therapist-patient relationship dynamic: given the risk that some patients are unsure of their own values, and given that some may welcome or be highly antagonised by a therapist that plays a more active role that challenges their values or flourishing conceptions, a preliminary conversation can establish the particular relationship dynamic a patient prefers – and, in particular, how much persuasion the patient can expect to be subjected to by the therapist.

To spell this out, consider the case below:

At age 19 years John developed a manic episode and was hospitalized. He was commenced on antipsychotic medication and therapy and since then, his ‘highs’ and periods of near normalcy have been punctuated by occasional depressive episodes. Now 40 and married, he has a unique and controversial understanding of what it means to “live life to the fullest”. He believes that experiencing the full spectrum of emotions, including his manic episodes, is essential for his creativity and entrepreneurial spirit. He argues that these episodes, although challenging, provide him with unique insights and a heightened sense of creativity, which he considers crucial to his identity as an artist and entrepreneur.

Despite medical advice, John resists taking mood stabilizers with antidepressant effects, not only because of the dampening effect on his energy and creativity but also because he feels these medications suppress a vital part of who he is. He views his manic states, albeit risky, as periods of intense productivity and inspiration.

John’s treating therapist is concerned about the risks associated with manic episodes, such as impaired judgment and potential for harm. However, John insists that these experiences are integral to his conception of flourishing, leading to a complex ethical and clinical dilemma.

In such a case, VBP would focus on ensuring that John is clear about the values driving his decision to prefer manic states for his creative and entrepreneurial endeavors. It would explore the trade-offs between his desire for unmediated emotional experiences and the potential risks of not using antidepressants or mood stabilizers regularly. VBP would also consider the values and preferences of John’s partner, acknowledging the interpersonal impact of his choices.

RLM would operate in a similar vein but might allow the psychiatrist to more actively engage with John’s controversial conception of flourishing. The psychiatrist could present arguments for why regular use of medication might be more beneficial in the long term, even if it means tempering some of John’s creative highs. This model would support a more assertive approach in recommending a treatment path while still ultimately leaving the decision with John.^7^

In both cases, John might in fact be unsure about his preferences and how they relate to his values. As mentioned, in such a case John may adopt his therapist’s own values as a way of cashing out the trade-offs, and the therapist might unwittingly oblige. Crucially, depending on John’s personality type and attitudes towards medicine or antidepressants in particular, there is a risk that, especially with RLM, he will cease seeing his therapist if he feels they will try to convince him of a particular course of action (such as continuing to take the antidepressant).

To minimize these chances, John and his therapist could have a brief conversation early on about John’s values and the relationship dynamic he prefers in therapy. The therapist can stipulate the conditions where they may transgress their agreement (e.g. due to legal or distributive justice-related reasons). Moreover, if there is a risk that a degree of unintended paternalism might seep into that initial conversation – whereby the therapist’s own relationship dynamic preference is unwittingly imposed – that conversation could be conducted by another ‘relaying’ therapist, or through a form to be filled out that the therapist can then examine before meeting the patient. This acknowledges the unequal power relations that exist between therapist and patient, and requires a degree of reflexivity on the therapist’s part to ensure that this ‘pre-work’ to agree on the relationship dynamic is properly and substantively conducted.

To be clear, we do not present this as an optimal or final solution to the question of what the appropriate therapist-relationship dynamic is. Instead, as noted at the outset of this section, this is intended as a concluding thought for future research on flourishing-oriented therapy. Given the pragmatic commitment of this approach, future empirical research would have to investigate trials of such a mechanism on patient uptake and outcomes, as well uncover potential hidden trade-offs that may be involved in terms of available funds and resources.

## Conclusion

We began this paper by noting that an exclusive focus on treating mental disorders can come apart from what is overall best for a patient’s well-being. We noted that calls for a more explicit focus on flourishing as an end goal of therapy face the question of what to do about patients who have unusual or controversial conceptions of flourishing. A dilemma appears to faces these calls: either have therapists adopt and prescribe a certain conception of the good life, or relegate the relationship to a simple consumer model. We argued that liberal models can help us think about this dilemma more constructively by adopting an alternative ‘democratic process’ that is not authoritarian nor purely consumerist. Critics of such approaches think they miss the point, confusing the process of uncovering and deliberating about values as an end in itself. This objection would straightforwardly apply to flourishing-oriented therapy that adopts the same liberal approach. However, we argued this need not be the case: there are pragmatic reasons to rely on such a process as the least bad option of the available (authoritarian or consumerist) alternatives. Indeed, we suggested one may not place much value on autonomy at all and yet still favour this approach. In the final section, we suggested that future research could consider the risk that paternalism may still seep into ostensibly interpretive or deliberative therapist-patient dynamics, and that in particular a deliberative approach may alienate certain patients who would prefer not to seek therapy at all than be confronted with arguments for a particular course of action. We concluded by suggesting that one possible solution is to use a preliminary conversation that enables patients to choose the particular relationship dynamic they prefer in their relationship with a therapist.

## Footnotes

1 The question of what to do in situations where patients lack capacity is perhaps the central question in mental health medical ethics, but in this paper we wish to focus on competent individuals who have unusual or controversial conceptions of flourishing.

2 Clinical practice is used to encompass both traditional psychiatric care (which entails both outpatient and inpatient management of psychiatric disorders using psychological and/or pharmacotherapeutic means in addition to which treatment may involve physical restraint, and interventions such as electroconvulsive therapy) and psychotherapeutic approaches that have their origins in psychodynamic models of the mind.

3 These are by no means the only two relevant examples, with ‘shared-decision making’ being another paradigm through which to describe and think about how clinicians and patients can approach medical decisions [23].

4 That is to say, just because a patient wants something, even if legal or harmless, a consumerist approach would still be limited by distributive justice constraints: there may be other justice-based reasons to refuse their requests that are due to limited resources.

5 Again, respecting patient autonomy here is importantly different from a purely consumerist consumer approach. There is not a process of eliciting and reasoning about value conflicts when one purchases an item from a supermarket, but there is when one is discussing treatment options that is nonetheless patient-centred.

6 *“Indeed it has been said that democracy is the worst form of Government except for all those other forms that have been tried from time to time.…”* [30] (Note, Churchill quotes another unnamed person in the attribution).

7 Unlike a traditional symptom-focused approach, where the primary goal is to manage or alleviate psychiatric symptoms within the boundaries of clinical and legal norms, the flourishing-oriented focus here goes a step further and actively engages with John’s conception of a good life, not simply to mitigate potential risks or discourage it outright, but to deeply understand its role in his life and sense of self holistically and integrate his values and experiences into his treatment plan.
